# The Norwegian dietary guidelines and colorectal cancer survival (CRC-NORDIET) study: a food-based multicentre randomized controlled trial

**DOI:** 10.1186/s12885-017-3072-4

**Published:** 2017-01-30

**Authors:** Hege Berg Henriksen, Hanna Ræder, Siv Kjølsrud Bøhn, Ingvild Paur, Ane Sørlie Kværner, Siv Åshild Billington, Morten Tandberg Eriksen, Gro Wiedsvang, Iris Erlund, Arne Færden, Marit Bragelien Veierød, Manuela Zucknick, Sigbjørn Smeland, Rune Blomhoff

**Affiliations:** 1 Department of Nutrition, Institute of Basic Medical Sciences, University of Oslo, Oslo, Norway; 2 Department of Gastroenterological Surgery, Oslo University Hospital, Ullevål, Norway; 3 Institute of Clinical Medicine, University of Oslo, Oslo, Norway; 4 National Institute for Health and Welfare, Helsinki, Finland; 5 Department of Digestive and Paediatric Surgery, Akershus University Hospital, Lørenskog, Norway; 6 Oslo Centre for Biostatistics and Epidemiology, Department of Biostatistics, Institute of Basic Medical Sciences, University of Oslo, Oslo, Norway; 7 Division of Cancer Medicine, Oslo University Hospital, Oslo, Norway

**Keywords:** Colorectal cancer, Disease-free survival, Overall survival, Time to recurrence, Cardiovascular disease-free survival, Comorbidity, Inflammation, Oxidative stress, Antioxidant-rich foods, Food-based dietary guidelines

## Abstract

**Background:**

Colorectal cancer survivors are not only at risk for recurrent disease but also at increased risk of comorbidities such as other cancers, cardiovascular disease, diabetes, hypertension and functional decline. In this trial, we aim at investigating whether a diet in accordance with the Norwegian food-based dietary guidelines and focusing at dampening inflammation and oxidative stress will improve long-term disease outcomes and survival in colorectal cancer patients.

**Methods/design:**

This paper presents the study protocol of the Norwegian Dietary Guidelines and Colorectal Cancer Survival study. Men and women aged 50–80 years diagnosed with primary invasive colorectal cancer (Stage I-III) are invited to this randomized controlled, parallel two-arm trial 2–9 months after curative surgery. The intervention group (*n =* 250) receives an intensive dietary intervention lasting for 12 months and a subsequent maintenance intervention for 14 years. The control group (*n =* 250) receives no dietary intervention other than standard clinical care. Both groups are offered equal general advice of physical activity. Patients are followed-up at 6 months and 1, 3, 5, 7, 10 and 15 years after baseline. The study center is located at the Department of Nutrition, University of Oslo, and patients are recruited from two hospitals within the South-Eastern Norway Regional Health Authority. Primary outcomes are disease-free survival and overall survival. Secondary outcomes are time to recurrence, cardiovascular disease-free survival, compliance to the dietary recommendations and the effects of the intervention on new comorbidities, intermediate biomarkers, nutrition status, physical activity, physical function and quality of life.

**Discussion:**

The current study is designed to gain a better understanding of the role of a healthy diet aimed at dampening inflammation and oxidative stress on long-term disease outcomes and survival in colorectal cancer patients. Since previous research on the role of diet for colorectal cancer survivors is limited, the study may be of great importance for this cancer population.

**Trial registration:**

ClinicalTrials.gov Identifier: NCT01570010.

**Electronic supplementary material:**

The online version of this article (doi:10.1186/s12885-017-3072-4) contains supplementary material, which is available to authorized users.

## Background

The incidences of colorectal cancer (CRC) are 5–10 times higher in Europe, North America and Oceania than in countries in Africa, south Asia and Central America [1], and the incidence in Norway is among the highest in the world [2]. Established risk factors for CRC are age, family history of CRC, inherited syndromes (Familial adenomatous polyposis, Lynch syndrome) and inflammatory bowel disease. In addition, several modifiable lifestyle-related risk factors are associated with CRC. Those include smoking, body fatness, abdominal fatness, diabetes, physical inactivity and an unhealthy diet (high consumption of alcohol, red and processed meat, and low consumption of foods containing dietary fibre) [3, 4]. World Cancer Research Fund (WCRF)/American Institute for Cancer Research (AICR) estimates that about 45% of all CRC cases could be prevented by improved lifestyle [3].

About 40% of CRC patients [5] have at least one concomitant disease (e.g. hypertension, cardiovascular disease (CVD), diabetes, chronic obstructive pulmonary disease or other malignancies) at the time of diagnosis and increased risk of developing additional comorbidities after CRC diagnosis [6–10]. These comorbid conditions may preclude or reduce effect of treatment, and consequently reduce disease-specific and total survival [8, 11, 12].

While it is well established that an unhealthy diet increases risk of CRC (e.g. see the latest update from World Cancer Research Fund, 2011 [4]) there are few studies that have focused on the effect of diet on disease outcomes and survival [13–15]. In paucity of data, health authorities in most countries recommend the same diet to CRC survivors (i.e. patients living with a CRC diagnosis, including those who have recovered) as to people without a cancer diagnosis [3].

Inflammation and oxidative stress are central underlying disease mechanisms in cancer and several other chronic diseases. Recent research suggests that there are two major molecular pathways leading to CRC, both of which involve inflammation and oxidative stress as major driving forces. The majority of CRC cases may be due to molecular events that result in chromosomal instability, while about 20-30% of CRCs are due to gene hypermethylation (called CpG island methylator phenotype (CIMP)) [16–18]. A large proportion of the CRC cases due to CIMP display microsatellite instability [18, 19]. In total, about 70 mutations in different genes have been identified as relevant for these two pathways to CRC, and it is assumed that each individual CRC tumor accumulates an average of 9 CRC pathogenic mutations out of this total pool of 70 mutations [16].

The heterogeneous pathogenesis of CRC comply with the hallmarks of cancer defined by Hanahan and Weinberg [20] and the cancer genome landscape as defined by Vogelstein et al [21]. Underlying these hallmarks of cancer, Hanahan and Weinberg proposed that genome instability and inflammation are two underlying driving forces [20]. These two processes or mechanisms are closely intertwined, since inflammation is a major cause of oxidative stress, and oxidative stress is a major cause of genome instability. Although inflammation and oxidative stress ultimately may be related to all CRC cases, the degree of inflammation and oxidative stress may vary significantly with the molecular signature present in the individual CRC patient [22].

In clinical trials and various models systems, we have identified a number of plant foods (e.g. berries, nuts, spices, coffee and specific fruits and vegetables) with the potential of dampening inflammation and oxidative stress [23–29]. Furthermore, a number of studies have also suggested that adherence to a prudent diet (e.g. Mediterranean diet) reduce inflammation and oxidative stress [30, 31]. We suggest that a prudent diet rich in specific plant-foods may be beneficial for CRC patients, especially those CRC cases with molecular signatures creating major inflammation and oxidative stress.

No intervention studies have investigated the role of diet in disease outcomes and survival in CRC-patients after diagnosis. Furthermore, no previous diet intervention study has focused on dampening inflammation and oxidative stress in this cancer population. This paper presents the background and design of a randomized controlled food-based diet intervention that examines the effects on disease outcomes and survival in CRC survivors. The diet intervention includes foods and drinks that have been suggested to dampen inflammation and oxidative stress. While specific anti-inflammatory and antioxidant-rich foods are emphasized in each food category, the complete intervention is fully in accordance with the prudent diet recommended by the Norwegian food-based dietary guidelines (NFBDG) [32] (i.e. a diet similar to the Mediterranean diet).

### Objectives

Outcomes are inconsistently defined in many clinical cancer trials [33, 34]. For the primary outcomes, we have used the proposed guidelines for outcomes as described by Punt et al [34]. The two primary outcomes are (to be assessed when all patients have completed 5, 10, and 15 years, respectively, of follow-up after baseline):

1. Disease-free survival (DFS) (events are defined as detection of local recurrence or metastasis or any second cancer or death from any cause)
2. Overall survival (OS) (event is defined as death from any cause)

Secondary outcomes are:

I. Time to recurrence (events are defined as detection of local recurrence or metastasis)
II. CVD -free survival (events of CVD (ICD-10; chapter I) or death from any cause)
III. CRC-specific survival (death due to CRC)
IV. Total cancer-specific survival (death due to CRC or any other cancer)
V. Inflammatory disease-specific survival (death due to inflammatory disease)
VI. Cardiovascular (CVD)-specific survival (death due to CVD)
VII. New morbidity of other diet-related chronic diseases (e.g. ischemic coronary heart disease, cerebrovascular disease, thromboembolic disease, type 2 diabetes, obesity, hypertension and chronic obstructive pulmonary disease)
VIII. Dietary intake and nutritional status
IX. Physical activity and function
X. Nutrition biomarkers (e.g., carotenoids, fatty acids, 25-hydroxy vitamin D)
XI. Body composition
XII. Anthropometric measures (e.g. weight, waist and hip circumference)
XIII. Biomarkers for inflammation and oxidative stress (e.g. isoprostanes, cytokines)
XIV. Transcription- and epigenetic profiles
XV. Biomarkers for cardiovascular disease, metabolic syndrome, type 2-diabetes, thromboembolic disease and cancer (e.g. blood pressure, total/LDL-cholesterol, HbA1c, CRP, IL-6, IL-10, TNFα)
XVI. Health related quality of life and fatigue

The secondary outcomes will be assessed after 5, 10, and 15 years and described in detail in subsequent reports. In addition, intervention effects on secondary outcomes VII-XVI will also be assessed at 6 months, 1 year and 3 years follow-up.

## Methods and Design

### Study design

**Fig. 1:**
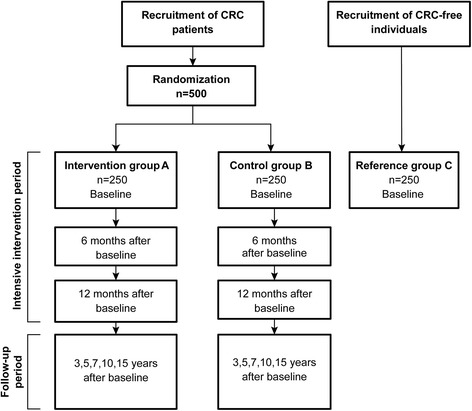
Study flow diagram

The CRC-NORDIET study is a multicentre, randomized controlled trial (RCT), with two parallel study arms. The intervention group receives an intensive dietary intervention and general advice on physical activity (see below), whereas the control group only receives standard general dietary advice and general advice on physical activity. Newly diagnosed CRC patients undergoing surgery are recruited to the study. In addition, an age-matched CRC-free reference group (will be published elsewhere) will also be included. The intervention starts 2–9 months after surgery (i.e. baseline), and consists of two periods: an intensive period that lasts 12 months, and a subsequent maintenance period which lasts an additional 14 years. Patients are invited to the study centre, situated at the Department of Nutrition, University of Oslo, at baseline, 6 and 12 months after baseline, and 3, 5, 7, 10 and 15 years after baseline. Additional follow-ups by regular mail, phone and e-mail, occur throughout the study. The study flow diagram is presented in Fig. 1. The design and handling of data of the CRC-NORDIET study is in fully agreement with the CONSORT statement [35].

### Patients and eligibility

Men and women 50 to 80 years of age with newly diagnosed primary invasive colorectal cancer (ICD-10 18-20), staged I-III (TNM-staging system [36]) are eligible for the study. The patients must be able to read and understand Norwegian and to provide a signed informed written consent. Patients unable to perceive information and understand the intervention due to diagnosed dementia, or altered mental status as well as patients participating in other RCTs in conflict with our trial are excluded from the study. Precise inclusion and exclusion criteria are presented in Table 1.

**Table 1:** Inclusion and exclusion criteria

Inclusion criteria	Primary adenocarsinoma colorectal cancer (ICD-10 C18-C20):
	C18 Malignant neoplasm of colon
	C18.0 Caecum
	C18.1 Appendix
	C18.2 Ascending colon
	C18.3 Hepatic flexure
	C18.4 Transverse colon
	C18.5 Splenic flexure
	C18.6 Descending colon
	C18.7 Sigmoid colon (sigmoid (flexure)
	C18.8 Overlapping lesion of colon
	C18.9 Colon, unspecified
	C19 Malignant neoplasm of rectosigmoid junction
	C20 Malignant neoplasm of rectum
	TNM stage I-III
	Age 50–80 years old
Exclusion criteria	Colorectal adenoma, carcinoid, abdominal carcinomatosis or sarcoma
	Unable to read and understand Norwegian
	Unable to perceive information and understand the intervention as such due to dementia or altered mental status
	Unable to follow the dietary intervention due to medical/clinical conditions e.g. total parental nutrition, permanently institutionalized
	Participation in another study in conflict with the intention of the CRC-NORDIET study

### Recruitment and randomization

Patients are recruited from Oslo University Hospital and Akershus University Hospital within the South-Eastern Norway Regional Health Authority. Screening for eligible patients is performed by research investigators in cooperation with hospital personnel by monthly reviews of surgery lists and medical records. Eligible patients are invited within 9 months from surgery.

Patients accepting the invitation sign an informed consent. Signed informed consent gives permission to the study personnel to take biological samples, perform physical measurements, and retrieve information from medical records, health registries and questionnaires. Information about storage of biological materials and use of individual data retrieved during the whole study for analysis and publishing purposes is also included in the informed consent letter.

Prior to baseline of the intervention, patients are randomized to either intervention group A or control group B in blocks of four. The random number sequence is computer-generated for each hospital. The person who generates the allocation sequence is neither the same person who determines eligibility nor the person that informs patients about their allocated study group. The patients are informed about the study group assignment at the baseline visit. Due to the nature of the intervention, neither the registered dietitians, nor the other research coworkers who meet the patients at the study centre, nor the patients themselves are blinded to group allocation.

### Intensive period of intervention

The CRC-NORDIET study offers an extensive intervention program for patients in group A, consisting of individual counselling on nutrition and physical activity, grocery discount cards, delivery of free food items, group meetings, printed materials, access to a CRC-NORDIET webpage and contact by telephone and e-mail. The patients in group B are offered the same individual counselling on physical activity as group A, as well as general group meetings. An overview of the intervention program and the instruments used are presented in Table 2 and Table 3, and in Additional file 1.

**Table 2:** Instruments used to facilitate compliance in intervention group A during the first 12 months

	Baseline (at study centre)	1 month (at home)	3 months (at home)	6 months (at study centre)	9 months (at home)	12 months (at study centre)
Nutritional counselling	Face to face individual	Phone call	Phone call	Face to face individual	Phone call	Face to face individual
Free-of-charge food	Delivered at the visit		Home delivery	Delivered at the visit	Home delivery	Delivered at the visit
Information/ courses	Folder with information on the study and the study instruments	Inspiration day and Cooking course				
Discount card	Discount card (25% discount on healthy foods)					
CRC-NORDIET Webpage/ e-mail	Login-restricted webpage access and e-mail communication					
Physical activity	Access to free training facilities (“Pusterommet”)					
Reports from non-biological measurements	Reports sent to the patients after every visit

**Table 3:** Instruments used in the control group during the first 12 months

	Baseline (at study centre)	1–12 months (1, 3, and 9 months at home, 6 and 12 months at the study centre)
Information/ courses	Folder with information on the study	Inspiration day
Physical activity	Access to free training facilities (“Pusterommet”)	
Reports from non-biological measurements	Reports sent to the patients after every visit	

#### Group A: diet intervention

Colorectal cancer patients experience different disease courses due to different stages at diagnosis, location of tumor, surgical procedure and adjuvant treatment. The diet intervention is therefore designed to meet the patients’ individual needs after surgery. In the initial phase, when symptoms related to cancer and cancer treatment are most common, the dietary focus is mainly on recovery and treatment of symptoms and progressive weight loss. Later, when symptoms and weight loss are treated and under control, and the disease conditions are more stable, the major focus is long-term disease-free living and secondary preventions. In this phase, we emphasize a diet which may dampen chronic inflammation and oxidative stress, fully in accordance with the NFBDG. A number of strategies are implemented to improve compliance to the recommended diet of the CRC patients in group A (see below).

##### The dietary recommendations in the CRC-NORDIET intervention

The NFBDG, published in 2011, was developed to prevent chronic diseases in the general population [32]. These guidelines are based on a comprehensive, systematic review of the evidence linking diet to risk of chronic diseases, including cancer. The guidelines do not provide a detailed diet plan, but define major aspects of the diet (Additional file 2). In the current study, the particular focus will be on the following NFBDG recommendations

1. daily intake of fruits, berries and vegetables (≥500 g/day)
2. weekly intake of 300-450 g fish
3. daily intake of 70-90 g wholegrains
4. limiting red and processed meat to maximum 500 g/week
5. keeping body weight within normal range of body mass index (BMI)
6. reduce intake of added sugar to < 10 E%
7. reduce salt intake to less than 6 g/day
8. achieving an average of at least 30 min of moderate (3–6 metabolic equivalents (METs)) physical activity per day or 150 min of moderate physical activity per week

The NFBDG can be implemented in different ways. For example, the recommendations of eating 500 g fruits, berries and vegetables every day may include different selections of individual foods, all compliant to the quantitative advice. However, not all of these foods may dampen inflammation and oxidative stress. Since inflammation and oxidative stress are ubiquitous as common basic pathogenic mechanism, we have selected to compose the intervention not only according to the NFBDG, but also by emphasizing those foods with strongest evidence for dampening low grade chronic inflammation and oxidative stress: We have identified foods and drinks that have high contents of redox-active compounds and/or have antioxidative effects individually or in combination in in vitro models, animal models, clinical trials and/or epidemiological studies [23–26, 28, 29, 37–56] (detailed list with references in Additional file 3):

- Drinks (e.g. coffee, black tea)
- Fruits and vegetables (e.g. onions, broccoli, tomatoes, carrots, pomegranates, garlic, oranges, olives)
- Berries (e.g. blueberries/bilberries, blackberries, and raspberries)
- Nuts (e.g. walnuts, almonds, and hazel nuts)
- Herbs and spices (e.g. thyme, oregano, clove, cinnamon, and rosemary)
- Whole grain (e.g. barley)
- Miscellaneous (dark chocolate)

Furthermore, we have also identified that the following foods and drinks may have anti-inflammatory effects individually or in combination in cell cultures, animal models, clinical trials and/or epidemiological studies (detailed list with references in Additional file 3):

- Coffee
- Fruits and vegetables (e.g. tomatoes, carrots, dog rose)
- Nuts (e.g. walnuts)
- Berries (e.g. strawberries, blueberries/bilberries, and blackberries)
- Whole grains
- Herbs and spices (e.g. thyme, oregano, and rosemary)

During the 15 year intervention period, these foods and drinks are gradually implemented in the advice to group A.

While these antioxidant- and phytochemical rich foods are advised as part of a balanced diet according to the NFBDG, patients were advised not to take any antioxidant supplements [55, 57].

#### Intervention strategies

The following instruments are used to facilitate compliance to the intervention in group A.

1. Individualized nutrition counselling by a registered clinical dietitian.

The nutritional counselling aims to meet the individual nutritional needs as well as educate the patients on how to change dietary habits in accordance with the NGBDG. In order to individualize the dietary advice, the registered clinical dietitian performs a comprehensive evaluation in each of the meetings (Fig. 1). The Patient-Generated Subjective Global Assessment (PG-SGA) tool [58] is used to assess nutritional status and nutritional impact symptoms. Weight and height measured the same day is used to calculate BMI, and current weight is compared with previous weight measurements to calculate weight changes. The presence of stoma is recorded as well as treatment status (i.e. whether or not the patient receives adjuvant treatment). Dietary intake is assessed by 24-h recall (at baseline). In addition, the registered clinical dietitian characterizes the patient’s current diet in relation to the NFBDG, and record use of supplements.

When the nutritional evaluation is completed, the patient receives dietary advice based on nutritional status and weight history. If the patient is malnourished or at risk of malnutrition (i.e. PG-SGA category B or C), dietary counselling primarily focuses on improving nutritional status by treating symptoms, ensuring an adequate energy and protein intake, and to prevent further nutritional deterioration. In terms of progressive weight loss, patients with PG-SGA B or C with BMI >20 are recommended to stabilize their body weight. Patients with BMI < 20 are recommended to increase their body weight within the range of a normal BMI, determined in the current study as BMI 20–27 for patients aged 50–80 years [59, 60]. Well-nourished patients (i.e. PG-SGA category A) with BMI >27 are recommended to decrease their weight within normal BMI range. The recommended change (weight gain or weight reduction) is set to maximum 3 kg in 6 months to ensure an optimal change in body composition.

If the patient is evaluated as well-nourished (PG-SGA A), the dietary counselling primarily focuses on the NFBDG. Examples of week menus are used to illustrate examples of foods and amounts to be eaten in adherence with the NFBDG. Food alternatives are given to adjust the week menu to the patient’s personal eating habits and preferences.

Motivation to change dietary habits in according to the NFBDG is recorded by asking whether the patient considers herself/himself to be either “very motivated”, “motivated”, “less motivated” or “not motivated”. When one of the last two categories is present, the registered clinical dietitian explores the potential to increase motivation by using techniques from Motivational Interviewing (MI) [61]. The degree of motivation (“very motivated”, “motivated”, “less motivated” or “not motivated”) is taken into account in each of the counselling sessions.

Each of the nutritional consultations is intended to result in a few dietary goals in agreement with the patient. It is emphasized that the patient defines her/his personal goals to increase the chances that he or she will succeed in changing dietary habits. The registered clinical dietitian aims at encouraging the patient to achieve these goals and the goals will be revised at next session. The telephone-based counselling in between the meetings at the study centre focus at monitoring the patient’s body weight status, dietary pattern according to the predefined goals and motivational status. In addition to the scheduled consultations at the study centre and by telephone, the patients have the opportunity to contact the registered clinical dietitian by e-mail during the entire intervention period. The same registered clinical dietitian follows the patient during the entire intervention period, when possible.

2. Discount card (25% discount on healthy foods)

The patients in the intervention group are offered a discount card from the retailer company, “Norgesgruppen”, which is Norway’s largest enterprise within the grocery market, with a market share of 40%. The discount card can be used within the first year of the intervention and gives a 25% discount on all fresh vegetables, fruit, berries and fish and on all food items marked with the keyhole symbol, which is used by the health authorities to label food that is considered the most healthy within its food category [62]. The discount card can be used in all food stores and supermarkets within “Norgesgruppen”.

3. Delivery of specific foods

The CRC-NORDIET is sponsored by several food producing companies with free food items, specifically selected in accordance with the anti-inflammatory and antioxidant-rich foods emphasized in this study, such as juice, garlic, tomato juice, fish, coffee, tea, cereals, whole grain bread, oils etc. At all visits to the study centre, the patients in group A receive a bag containing a mixture of these food items. In addition, they receive a box with free food items delivered to their homes two times during the intensive period of the intervention.

4. CRC-NORDIET website

The patients in the intervention group get access to a login-restricted, dynamic website with detailed information about the NFBDG, portion sizes of recommended intake of fruits and vegetables and whole grain, food recipes, examples of week menus, dietary advice for treatment-related symptoms and advice on physical activity. In addition, information about the CRC-NORDIET study and contact information for the study organizers are given. The website is continuously updated.

5. Printed materials

The patients in the intervention group receive printed materials at the first visit to the study centre and at all follow-ups to ensure that also patients who do not use the internet get all relevant information.

6. Cooking course

During the first 6 months of the intervention, each patient in group A is offered a one-day cooking course. This course is led by a registered clinical dietitian who follows a protocol developed for the CRC-NORDIET intervention. The aim of the cooking course is to give the patients practical experience in making healthy dishes and to introduce healthy choices when shopping for food. The course consists of a one hour lecture on the NFBDG and how to implement these guidelines in daily cooking. All recipes can also be found on the CRC-NORDIET web site.

7. Physical activity

The CRC-NORDIET study has an agreement with “Active against cancer” [63], a non-governmental non-profit organization founded in 2007. The organization operates a free training studio (“Pusterommet”) for cancer patients at several hospitals in Norway. The physical therapists working at these studios are instructed to give individualized advice for exercises during and after cancer treatment. The CRC-NORDIET patients are encouraged to utilize this offer.

Moreover, the CRC-NORDIET patients are advised to practice moderate physical activity for at least 30 min per day, or 150 min per week, and they receive a booklet on how to be physically active in daily life. In addition, they are recommended to use local facilities, including swimming pool, health training centres and walks in their neighbourhoods.

8. Inspiration day

The patients in Group A are invited to an inspiration day within the first 6 months of the intervention. The day opens with a 45 min lecture about the aim and background of the CRC-NORDIET study by the project leader, with special focus on the NFBDG. The patients are shown examples of different portion sizes of fruits and vegetables, nuts, whole grain products, the food-dish-model, and have the opportunity to talk to registered clinical dieticians. The last part of the inspiration day focuses on physical activity, and starts with a lecture about physical activity incorporated in daily life. The patients also meet the physical therapists from “Pusterommet”. The meeting ends with a lunch and a quiz about physical activity, and each patient receives a pedometer as an incentive to be physically active.

9. Written reports

The patients receive reports from the non-biological samplings (e.g. anthropometric measurements and blood pressure, described in detail in the following section) performed at the three time points during the intensive intervention period (baseline, 6 and 12 months after baseline), as well as a one-year report showing the development during the last year. Reports from the physical activity monitors are given to the patients after the first intensive year of intervention.

#### Group B: control group

1. Physical activity

Patients in the control group receive the same basic advice on physical activity as well as free access to the training studio as patients in the intervention group (see above).

2. Inspiration day

The inspiration day is structured identically as for group A, except for the session focusing particularly on diet, which is excluded in the inspiration day for group B.

3. Dietary information

The patients in group B receive a booklet with basic dietary advice at baseline. In contrast to the intervention group, the control group receives no individualized dietary advice adapted to their eating habits and preferences. If they seek counselling concerning symptoms related to cancer or cancer treatment, the registered clinical dietitians provide dietary advice based on information from booklets and other printed materials already available in the hospitals. This information and dietary advice is considered as part of the standard care.

4. Written reports

The patients in group B receive written reports similarly as group A after all visits during the intensive intervention period.

### Moderate intervention during maintenance period (year 2–15)

During the maintenance period, which starts after the first intensive year and lasts for 14 years, both groups receive reports (e.g. anthropometric measurements and blood pressure) following every visit at study centre (year 3, 5, 7, 10 and 15).

The patients in group A are invited to an inspiration day every year during moderate period of intervention. The aim of these meetings is to maintain the focus on foods dampening inflammation and oxidative stress and the NFBDG, and to encourage the patients to continue following the guidelines in a long-term perspective. In addition, group A are offered dietary counselling at each visit at the study centre, as well as a telephone counselling by the registered clinical dietitians once a year. They also have access to the CRC-NORDIET webpage which is continuously updated with information and encouragements (e.g. recipes, nutrition information, motivational tips and relevant popular reports from nutritional sciences) until the end of study participation. An overview of the instruments used during the maintenance period is presented in Table 4.

**Table 4:** Instruments offered to the respective groups during maintenance period of intervention

Instruments	Time points	
	2, 4, 6, 8, 9, 11, 12, 13, 14 years	3, 5, 7, 10, 15 years
Dietary counselling at study centre (Group A)		X
Dietary counselling by telephone (Group A)	X	X
Inspiration day with extended diet session (Group A)	X	X
CRC-NORDIET Website/e-mail (Group A)	X	X
Reports from non-biological measurements (Group A and B)		X

### Assessment of primary outcomes

Several registries and medical records will be used for assessment of primary outcomes. The registries and time points for primary outcome assessment are summarized in Table 5.

**Table 5:** Data source used to assess primary outcomes

Outcome	Instrument	5 years after baseline	10 years after baseline	15 years after baseline
DFS	Colorectal Cancer Registry of Norway, Cancer Registry of Norway, Cause of Death Registry in Norway, Norwegian Patient Registry, Norwegian Prescription Database	X	X	X
OS	Cause of Death Registry in Norway	X	X	X

*DFS* disease-free survival, *OS* overall survival

### Questionnaires, biological samplings and measurements

Group A and Group B are undergoing equal regimes of measurements and biological samplings at all visits (Additional file 4). All patients are also asked to complete several questionnaires regarding demographic information, dietary intake, health status and physical activity (described below) (Additional file 4). The questionnaires administered at baseline of intervention are also completed at 6 months and 12 months follow-up. After the first year, the patients are invited to the study centre for questionnaires, biological samplings and measurements 3, 5, 7, 10 and 15 years after baseline. In addition to the visits to the study centre during the maintenance period, finger prick blood sample equipment (dried blood-spot cards) and questionnaires are sent to the patients’ home at certain time points and subsequently returned to the study centre.

#### Demographic information

A short questionnaire is used to assess demographic characteristics including age, gender, marital status, ethnicity, level of education, working status, family history of CRC or other type of cancer.

#### Assessment of dietary intake

##### Semi-quantitative food frequency questionnaire (FFQ)

The semi-quantitative 282-item FFQ used in CRC-NORDIET is designed to assess habitual diet over the preceding year, including both frequency of intake and portion sizes. The FFQ is described and validated elsewhere [64, 65].

##### Compliance questionnaire

The compliance questionnaire is a semi-quantitative short 63-item FFQ, developed within this study and designed to assess the dietary intake (grams per day) and physical activity (minutes per day) for the last 1–2 months. The questions correspond to the food groups and the recommendations regarding physical activity of the NFBDG. The questionnaire will be validated within the first period of study.

##### Food records

Food intake is recorded by using a 7-days weighed food record. The patients are provided with a food diary and a digital scale, and are instructed on how to weigh and record all foods and beverages consumed during a period of seven days. The food diary include all days of a week, and can either record seven consecutive days or be divided into two periods of three and four days within two weeks. The food records are performed in a subgroup of patients (will be published elsewhere).

##### 24-h recall

A registered clinical dietitian performs a 24-h recall at baseline by asking the patients in the intervention group in details about the intake of foods and drink during the past 24-h period. The 24-h recall is performed only in intervention patients since it is an integrated part of the nutritional counselling.

#### Assessment of physical activity and function

##### Recording of daily physical activity

The physical activity monitor SenseWear Mini Armband (BodyMedia, Pittsburgh, Pennsylvania, USA) [66] is used to record daily physical activity, inactivity and energy expenditure during seven consecutive days among all patients in both study arms at all visits. The armband monitors physiological data such as heat flux, galvanic skin response, 3-axis accelerometer and skin temperature. All data are retrieved from the armband to the computer with the SenseWear Professional Software [66]. The participant are instructed how to use the armband, and return it in a stamped envelope to the CRC-NORDIET study at the end of the test period. The armband is pre-programmed with the co-predictors such as weight, height, age, gender, smoking status (smoker/non-smoker) and placed around the non-dominant arm.

##### Self-reported physical activity

The patients are asked to complete a questionnaire regarding frequency, intensity and duration of their daily physical activity, as well as duration of sedentary time. These questions are based on the questionnaire from the HUNT 3 study in Norway [67].

##### 6-min walking test

Patients are invited to a 6-min walk test (6MWT) at several time points. The test is performed indoors, along a long, flat, straight, enclosed corridor with a hard surface. The walking course is 30 m in length, and cones mark the turnaround points. A countdown timer (or stopwatch) is used to record the time of the test. Prior to the test, the researcher measures the blood pressure of the patient. In addition, the pulse is monitored before, during and after the test. The patients are asked to grade its level of shortness of breath and the level of fatigue by using the Borg scale 6-20 before and after the test. Total length of walking (in meters) is recorded during 6 min of time.

##### Sit-to-stand test

The test is performed by the use of a straight back chair with a solid seat at the height of 44 cm. The patients are instructed to sit on the chair with arms folded across their chest, and then to stand up and sit down as quickly and frequently as possible within 30 s, keeping both arms folded across the chest. The number of stands during this period is counted.

##### Handgrip strength

Hand-grip strength is measured by the MAP 80 K1 Hand grip dynamometer (KERN & SOHN GmbH, Balingen, Germany) and measured as described in the manufacturer’s protocol [68]. The maximal strength of hand grip (kg) is recorded. For women and men, a 40 kg- and 80 kg-spring is used, respectively. The grip strength is measured with one punch and repeated three times on both hands. The maximum hand-grip strength on both left and right hands are recorded.

#### Assessment of nutritional status

##### Patient-Generated Subjective Global Assessment (PG-SGA)

Nutritional status is measured by using the scored PG-SGA [58], a nutritional assessment tool specifically developed and validated for cancer patients. A translated (Norwegian) version is used. Both the global categories well-nourished (A), moderate malnourished (B) and severe malnourished (C), as well as the numerical scoring system are used to characterize the nutritional status.

#### Anthropometric measurements

##### Body weight

Body weight (kg) is measured by using a non-slip Marsden M-420 Digital Portable Floor Scale (Marshden, Rotherham, South Yorkshire, United Kingdom) or a digital wireless measuring station for height and weight, Seca 285 (Seca, Birmingham, United Kingdom) [69]. Measurements are performed with light clothes and without shoes. Body weight is recorded with 2 decimals and the kind of clothing is recorded.

##### Height

Height (cm) is measured using either a mechanical height rod (Kern MSF- 200, [68]) or a digital wireless stadiometer (Seca 285 [69]). The height is recorded with one decimal precision.

##### Waist and hip circumference

Waist circumference is measured at the midpoint between the lower margin of the last palpable rib and the top of the iliac crest, whereas the hip circumference is measured around the widest portion of the hips. Waist and hip circumference are used to calculate the waist hip-ratio (WHR) which is a well-established indicator of abdominal fatness [70].

#### Body composition analysis

##### Bioelectrical impedance analysis (BIA)

BIA is performed under standardized conditions by the use of BIA 101 (SMT Medical, Würzburg, Germany) that applies a current of 0,8 μA at a frequency of 50 kHz. Four skin electrodes are placed on hand and foot of the patients when lying in supine position. All measurements are conducted on the patients’ right side as instructed by the manual. Resistance (Rz) and reactance (Xc) are used in appropriate and validated equations to calculate body composition compartments such as fat mass, fat free mass and muscle mass. In addition, BIA is also performed with Seca mBCA515 (Seca, Birmingham, United Kingdom) [71]. Patients carrying a pacemaker are excluded from the BIA measurements.

##### Dual-energy x-ray absorptiometry (DXA)

The Lunar iDXA (GE Healthcare Lunar, Buckinghamshire, United Kingdom) is used to measure bone mineral density and body composition, including quantification of visceral fat.

##### Computed tomography (CT)

CT images taken routinely for clinical purposes are used for body composition analysis, i.e. quantification of fat (visceral, subcutaneous and intermuscular adipose tissue) and skeletal muscle. The images are analysed using the Slice-o-matic software, version 4.3 (Tomovision, Montreal, Canada). The third lumbar vertebra (L3) is chosen as standard landmark since skeletal muscle, lean tissue mass and adipose tissue at this level are significantly correlated to whole-body tissue in healthy adults [72].

#### Blood pressure

Blood pressure (BP) is measured with the digital blood pressure patient monitor Carescape V100 (GE Healthcare, Fairfield, USA) and performed by trained staff following the clinical procedure as described by the manufacturer [73]. After a 5 min resting period in a silent room, BP is measured four times on the non-dominant arm with intervals of one minute.

#### Biobank

A variety of biological samples will be collected at different time points during the study and will be used for the purposes of measuring surrogate outcomes, biomarkers of food intake and for identification of phenotypes associated with different responses to the intervention.

##### Venous blood samples

Overnight fasting blood samples are taken between 07.30 and 10.30 at the study centre by a trained technician. BD Vacutainer® (Becton, Dickinson and Co, Franklin Lakes, NJ, USA) tubes are used to collect ethylene diamine tetraacetic acid (EDTA) samples (no. 367861 and 366643), serum samples (no 368774), lithium heparin samples (no 367526), and citrate samples (no 369714).

Serum tubes are placed in room temperature for 30 min. Serum, EDTA and heparin samples are centrifuged at1500 *g*, 10 min, 15 °C. Serum, plasma and red blood cells are aliquoted, and immediately stored in at −80 °C until further analysis. Whole blood from EDTA samples are also aliquoted for e.g. DNA extraction and DNA damage/repair analysis The buffy coat from the heparin samples are either frozen at −80 °C for later analysis or used to obtain isolated peripheral blood mononuclear cells (PBMC) through Percoll centrifugation. The isolated PBMCs from heparin samples are used for *ex vivo* experiments. Two citrate tubes are kept 1 h respectively at 4 °C and room temperature before centrifugation (2500 *g*, 15 min, 4 °C) to obtain core plasma, plasma and red blood cell aliquots that are stored at -70 °C. One citrate tube is centrifuged (2500 *g*, 15 min, 4 °C) within 30 min of sampling, and core plasma is stored at - 80 °C for further analysis of thromboembolic factors. The citrate buffy coats are used to obtain isolated PBMCs for the study of DNA repair and DNA damage. PAXgene Blood RNA Tubes (cat.no 762115, PreAnalytiX, Hombrechtikon, Switzerland) are used as source for total blood RNA. The tubes are kept 2 h at room temperature before they are frozen at -20 °C for 24 h and subsequently transferred to −80 °C until time for RNA isolation.

##### Isolation of buffy coats from EDTA samples

The EDTA buffy coats are re-solved in 9% NaCl (cat.no 586564, B.Braun Melsungen AB, Melsungen, Germany) solution before added on top of 4 ml Lymphoprep (cat.no 1114545, Axis-Shield, Oslo, Norway) in a 15 ml tube for centrifugation (20 min RT 400 g) to isolate PBMCs which are further used for a chromatin crosslinking procedure. The crosslink procedure for preparing the cell pellets for ChIP-chip analysis are performed as follows: Firstly, PBMCs are allowed to crosslink with 1% formaldehyde (final concentration) for 10 min at room temperature, adding glycine (0.125 M) for 10 min at room temperature to stop the crosslinking process. After washing the cell pellets twice with 10 mL of ice-cold 1 × PBS the pellets are immediately stored in 2 ml plastic tubes at −80 °C until proceeding further with protocols for Chip-on-Chip analysis at a later time point.

##### Finger prick blood samples

Finger prick blood samples for analysis of e.g. biomarkers of dietary intake, oxidative stress and oxidative damage are collected by the dried blood spots (DBS) method as previously described [74]. DBS cards (2 cards per patient) are allowed to dry in room temperature for 2 h and are frozen at −80 °C in airtight aluminium bag with a desiccant until further analysis.

##### Urine samples

Biomarkers of food intake, oxidative stress and other risk factors related to the progression of CRC will be measured in urine. Urine samples are collected from a subpopulation several times during the intervention by the methods as previously described [75–77].

##### Faeces samples

Microbiotica and biomarkers related to CRC will be measured in faeces samples which are collected from a subpopulation several times during the intervention. The patients will receive a specific faeces sample tool kit and are asked to collect the sample at home and mail it to the study centre. Sampling and analysing of the faeces samples will be performed by following the procedure as described by Naseribafrouei [78].

##### Tumour tissue

Molecular signatures in CRC tumours that are linked to inflammation, oxidative stress and energy balance have been shown to predict response to lifestyle intervention. Characterization of tumor markers will be performed by immunohistochemistry, PCR, sequencing and q‐PCR (to be published elsewhere). Furthermore, we will study whether tumor markers predict response to the dietary intervention. Samples of tumor tissue are collected at surgery in collaboration with the hospitals. Molecular signature data are also obtained from the CRC biobank project at the Oslo University Hospital.

#### Oral glucose tolerance test

Prior to the oral glucose tolerance test, the patient is fasting for at least 8 h. Blood samples (serum and PAX tubes) are taken, and blood glucose is measured with a blood glucose meter [79]. The patients are asked to drink 75 g of glucose (D (+)-Glucose (product number: 1370485000, Merck-Millipore Corp, Darmstadt, Germany) in 4 dl of boiled water. The glucose liquid is expected to be consumed total within 5 min. Blood samples will be taken after 2 h. Exclusion criteria for oral glucose tolerance test are Diabetes Type I, use of insulin and/or fasting blood glucose level exceeding 10 mmol/l.

#### Health related quality of life and fatigue

Quality of life will be self-reported and measured using the generic, multi-purpose-form questionnaire for Health related quality of life (HRQOL) called Short form (SF) health survey consisting of 36 items (SF-36) [80]. The 36 items are categorized into eight multi-item scales; 1) physical functioning, 2) role physical, 3) bodily pain, 4) general health, 5) vitality, 6) social functioning, 7) role emotional and 8) mental health as well as a single-item measuring health transition during the last year. The data will first be standardized in order to compare results across studies [80] and then recoded according to a syntax developed by Loge et al [81].

A validated generic fatigue questionnaire (FQ) is used to assess the patients subjective fatigue status (11 items) and the duration and extent of fatigue (2 items) [82]. The FQ asks about fatigue symptoms experienced during the last month compared to how the subject felt when she/he was last feeling well [82–85]. Each item has four response-choices [82]. The scoring of each response is based on a Likert- (0, 1, 2, 3) and a dichotomized (0, 0, 1, 1) scale. The latter is only used for case definition. The total sum of the Likert-scores is designated total fatigue (TF) where higher scores imply more fatigue.

#### Assessment of new morbidity of diet-related chronic diseases and adverse events

New morbidity of diet-related chronic diseases arising after CRC diagnosis (e.g. ischemic coronary heart disease, cerebrovascular disease, thromboembolic disease, diabetes, hypertension and chronic obstructive pulmonary disease) will be collected from the national health registries in Norway, a comorbidity questionnaire developed for this study designed to assess comorbidity based on data from the third Norwegian population health study (HUNT 3) [67], and from medical records. These data will be supplemented by data on drug use from the Norwegian Prescription Register. Adverse events are recorded based on the Common Terminology Criteria for Adverse Events (CTCAE, Version 4.0) [86].

### Sample size

#### Calculation for primary outcomes

The sample size calculations are based on assuming a Weibull distribution for the survival times in both arms. We further assume a constant hazard ratio for the intervention effect over time and that we have the same follow-up of 5, 10, or 15 years, respectively, for all patients. Sample sizes required to achieve a statistical power of 80% and significance level of 5% were calculated with computer simulations using the spower function in R (version 3.2.0) package Hmisc version 3.17–0. Survival rates in the control group [2, 87] and expected reduction in mortality rates in the intervention group are taken from the literature (see Discussion, [88–93]). With a 68% 5-year OS in the control group, we have 80% power to detect a 25% reduction in mortality due to the intervention (corresponding to a hazard ratio of 0.71). The required total sample size is then 500 (250 in each study group) (Table 6).

**Table 6:** Sample size in each group (n) and hazard ratios (HRs*) for selected scenarios of reduction in mortality by intervention. The power is 80% and significance level 5%

	Reduction in mortality by intervention			Survival rates in the control group
Primary outcome	*20%*	*25%*	*30%*	
	n (HR)	n (HR)	n (HR)	
*DFS*				
5 years	320 (0.753)	190 (0.696)	140 (0.641)	0.59
10 years	240 (0.716)	140 (0.655)	110 (0.597)	0.41
15 years	180 (0.680)	120 (0.616)	90 (0.557)	0.29
*OS*				
5 years	390 (0.767)	250 (0.712)	160 (0.658)	0.68
10 years	280 (0.732)	180 (0.673)	130 (0.616)	0.48
15 years	210 (0.695)	140 (0.632)	100 (0.574)	0.34

* HR = hazard ratio of intervention versus control, which corresponds to the assumed survival rate in the control group and assumed reduction in mortality by intervention

*DFS* disease-free survival, *OS* overall survival

Moreover, sample size calculation based on 25% reduction in events of DFS after 5 years of surgery (59% 5-year DFS in the control group), we have 80% power to detect HR of 0.70, with 190 patients in each group (Table 6).

#### Stratified and subgroup analysis

All of the power calculations are based on a heterogeneous population of CRC patients.

Since post-surgery treatment may vary, and colon versus rectum cancer may respond differently to the diet intervention, we will also perform stratified statistical analysis. It is not known whether treatment effects are different in these subgroups. These stratified analyses will be conducted with primary outcomes at the later time-points in the study and at all time-points to assess mean differences between the control and the intervention groups in biomarker analysis, as these data normally require fewer patients per group.

#### Statistical analysis

Data will be analysed using SPSS (IBM SPSS Statistic 22). For the survival outcomes (primary outcome 1 and 2, and secondary outcomes I-VI) tests will be performed to compare survival rates between the control and intervention groups at 5, 10, and 15 years after baseline. Survival probabilities will be estimated with the Kaplan-Meier method. Cox proportional hazards models will be used to identify prognostic and predictive biomarkers for survival outcomes.

For non-survival secondary outcomes, parametric or non-parametric tests for two-group comparisons will be used to assess group differences at individual time-points. In addition, mixed effect models for longitudinal data and regression models will be used to evaluate association and change over time in dietary intake, nutritional status, body composition, molecular tumor characteristics, physical function and activity, quality of life, fatigue and treatment related outcomes and to examine differences between the intervention and control groups. All statistical tests are performed as two-sided tests. Effects are considered statistically significant if *p <*0.05.

## Discussion

The primary aims of the CRC-NORDIET are to study whether a healthy diet rich in anti-inflammatory and antioxidant-rich foods and based on the NFBDG can improve DFS and OS in CRC patients. To our knowledge, this is the first randomized controlled trial designed to investigate the effect of a dietary intervention on these outcomes in CRC patients, and to investigate the potential role of diet in dampening of inflammation and oxidative stress in these patients. The multiple strategies used to achieve compliance to the intervention during the first year, followed by the 14 years maintenance and follow-up period make the design of this intervention unique.

Data on role of diet on disease outcomes and survival in CRC survivors is limited. To date, several cohort studies, but no RCTs have investigated the effects of food-based dietary interventions on these outcomes. Data from a US cohort study with stage III colon cancer patients suggest that high intakes of red and processed meat, fat, refined grains and dessert, i.e. a Western dietary pattern, after diagnosis are associated with a significantly reduced disease-free and OS [13]. Similar findings are reported in a Canadian cohort study with CRC patients staged I-III, where patients with the highest intake of processed meat the previous year before diagnosis had an 82% increased risk of recurrence or death compared with patients with the lowest intake [15].

Prospective cohort studies have consistently reported that physical activity after colorectal cancer diagnosis reduces risk of mortality. In a meta-analysis of six prospective cohort studies, including 7522 CRC survivors, the authors observed that the most physical active survivors had a 42% lower risk of total mortality compared to those who were least active. The risk reduction of cancer-specific mortality was 39% [94]. No RCT has so far confirmed that physical activity impacts mortality in CRC survivors.

Interventions designed to investigate the effect of diet separated from other lifestyle factors (smoking, physical activity, weight regulation) are needed in order to investigate whether there is a causal relationship between diet and survival as well as disease-related outcomes. Our intervention is intended to change the dietary habits towards a diet in agreement with the NFBDG. These dietary guidelines are developed to prevent chronic diseases, including cancers, in the general population. Several large cohort studies have shown that there is a consistent inverse association between adherence to cancer prevention guidelines and cancer-specific and all-cause mortality [89, 95]. Among cancer survivors, reduction in total mortality between highest versus lowest score in adherence to diet recommendations has been documented in five different cohort studies, ranging from 24% to 36% (follow-up period from 3.7 to 13.6 years) [88–93]. Association of adherence to American Cancer Society guidelines and reduction in death attributed to cancer has been shown to be 25 and 26% in men and women, respectively [89]. Hastert et al documented an association of adherence to the WCRF/AICR guidelines and reduction in cancer-specific mortality of 61% in respondents with the highest compared to the lowest WCRF/AICR score (follow-up time of 7.7 years) [95].

Furthermore, NFBDG include advice regarding red and processed meat, dietary fibre, dairy products and garlic, all of which are related to risk of CRC. Whether these dietary factors also may have effect on survival and disease outcomes, remain unclear. With improvement in cancer survival, these perspectives are increasingly important. The main objective of the present study is to test if diet will improve survival and cancer-related outcomes, mediated through reduced inflammation and oxidative stress. To strengthen this assumption, we have chosen to select specific foods within the NFBDG which have been identified as anti-inflammatory or antioxidant-rich in previous preclinical and clinical studies [23–29, 37–39, 41–48, 53, 55, 96].

Four aspects that may be of importance for achieving lifestyle changes and facilitate compliance to the intervention: 1) timing of intervention, 2) choice of motivational approach to achieve lifestyle changes, 3) duration of intervention, and 4) use of incentives and methods to achieve sustainable changes. Previous studies have shown that cancer patients in general are particularly motivated to change dietary habits at the time of diagnosis, often reported as the teachable moment [97–99]. Interventions designed to include this teachable moment have shown to be successful [97–99]. In our trial, we introduce the dietary intervention within a few months from surgery, thereby expecting we reach the patients within the time frame of this teachable moment. Furthermore, principles from MI [61] are implemented in each of the dietary counselling sessions by trained registered clinical dietitians. Previously published trials that have succeeded in changing lifestyle behaviours in cancer patients are based on theoretical frameworks and theories, including social cognitive behaviour therapy and use of MI. It is emphasized that the patient defines her/his own goals to increase the chances that he or she will succeed in changing dietary habits. We suggest that it is important to focus on a few realistic goals at the time, instead of aiming at changing the whole diet immediately. In addition, follow-up by the same registered clinical dietitian during the entire intensive intervention period may be of importance for the commitment to the intervention goals.

In order to increase the chances of sustainable lifestyle changes and compliance to the intervention, our intervention consists of a one year intensive period and a subsequent maintenance period which lasts for until 14 years. Taking into account that the teachable moment may vary among the patients and it may take time to establish new sustainable dietary habits, the inclusion of a maintenance period will probably be beneficial with regard to an increased long-term adherence to the intervention. Previous published trials that have failed in compliance from the patients may have had too short time frame of diet intervention.

Different strategies are reported to be effective in promoting lifestyle changes in cancer survivors [14, 97, 98, 100, 101]. Interventions focusing on individual counselling [14, 98, 100], and also interventions with a mixed strategy of individual in-person counselling, telephone counselling and mailed materials [97, 101] have been shown to be effective in health behaviour change among CRC survivors. Thus, during the first year, the CRC-NORDIET study offers individualized counselling, free foods, a discount card on healthy foods, access to a login-restricted web page, printed materials, cooking courses and inspiration day, which may all be effective incentives to follow the NFBDG.

In placebo-controlled RCTs, an intervention is tested by comparing one group of individuals who receive the intervention with a control group who receives a placebo. This type of placebo-controlled RCT is most often not possible when studying food-, or exercise-based interventions, since placebo-foods or placebo-exercise do not exist. In addition, no food based intervention can be analysed thoroughly without considerations regarding energy intake and energy expenditure. We have therefore selected to give the intervention group and the control group the same advice on physical activity. We include careful monitoring of physical activity to control for any confounding effects of physical activity. Of ethical reasons, we also include standard dietary advice (i.e. standard clinical care) in the control group, as well invitations to group meetings and feed-back reports on health status. Thus, while the control group in the present study is not identical to a placebo group, this particular study design was used in order to isolate the effect of diet intervention on CRC patients, and to reduce drop-outs from the control group, which is a common concern in long-term intervention trials.

Sample size estimation is not straightforward in RCTs with complex diet intervention and long term hard outcomes. This is especially the case when no similar trials have been previously published. By using the best available information from scientific literature and Norway Cancer Registry on survival rates in the control group and expected reduction in mortality rates in the intervention group, we have performed power calculations on the two primary outcomes after 5, 10 and 15 years after baseline. We conclude that 250 patients in each group would give us a reasonable chance (at 80% power) to detect any significant effects (see Methods and Design section for details) after 5, 10 and 15 years.

In a similar study, testing the effects of two different 6 months adjuvant cytostatic protocols (i.e. the MOSAIC study [102]), 2246 patients who had undergone curative resection for stage II and III colon cancer, were recruited. After a median follow-up for 38 months, fewer cancer-related events was observed in the alternative treatment group compared to the standard treatment group (HR 0.77, *p =* 0.002). The main reason for the lower number of patients required in our CRC-NORDIET study compared to the MOSAIC study is due to an older population with more expected events (50–80 years versus 19–75 years) and a longer follow-up time (10 and 15 years versus 3 years).

We have also performed a number of power estimations on secondary outcomes (data not shown). In general, the CRC-NORDIET study is expected to have enough statistical power to detect significant effects in a majority of these intermediate outcomes (to be published in relevant reports). These power calculations on primary and secondary outcomes are also supported from the RCTs with physical activity intervention in CRC and breast cancer patients; Friedenreich and Courneya detected significant effects on intermediate outcomes (e.g. inflammation biomarkers) as well as disease outcomes in RCTs with 200–250 patients per group [103–105].

### Conclusion and perspectives

The CRC-NORDIET study investigates whether a diet aimed at dampening inflammation and oxidative stress and in full accordance with the NFBDG will improve survival and disease outcomes in CRC patients. This RCT is unique in several aspects related to the interventions as well as outcomes. Since previous research on the role of diet for CRC survivors is limited, the study is important in order to improve health outcomes and survival in this population.

## Additional files

Additional file 1: The CRC-NORDIET intervention program (DOC 42 kb)
Additional file 2: Summary of the 13 recommendations of the Norwegian food-based dietary guidelines (NFBDG) (directly translated from the NFBDG) (DOCX 23 kb)
Additional file 3: Detailed list of foods and drinks with high contents of redox-active compounds and/or antioxidative effects (DOCX 22 kb)
Additional file 4: Questionnaires, biological samplings and measurements (DOC 43 kb)

## Abbreviations

6MWT: 6-min walking test
ACS: American Cancer Society
AICR: American Institute of Cancer Research
BIA: Bioelectrical impedance analysis
BMI: Body mass index
BP: Blood pressure
CIMP: CpG island methylator phenotype
CRC: Colorectal cancer
CRP: C-reactive protein
CT: Computerized tomography
CTCAE: Common terminology criteria for adverse events
CVD: Cardiovascular diseases
DBS: Dried blood spots
DFS: Disease-free survival
DNA: Deoxyribonucleic acid
DXA: Dual-energy x-ray absorptiometry
EDTA: Ethylene diamine tetraacetic acid
FFQ: Food frequency questionnaire
FQ: Fatigue questionnaire
HbA1c: Glycated hemoglobin A1c
HR: Hazard ratio
HRQOL: Health related quality of life
HUNT: Helseundersøkelsen i Nord-Trøndelag
ICD: International classification of diseases and related health problems
IL: Interleukin
L3: Third lumbar vertebra
LDL: Low density lipoprotein
METs: Metabolic equivalents
MI: Motivational interview
NFBDG: Norwegian food-based dietary guidelines
OS: Overall survival
PBMC: Peripheral blood mononuclear cell
PG-SGA: Patient-Generated Subjective Global Assessment
RCT: Randomized controlled trial
RT-PCR: Real-time quantitative reverse transcription polymerase chain reaction
SF-36: Short form-36
TNFα: Tumor necrosis factor alpha
TNM: Tumor Node Metastases
WCRF: World Cancer Research Fund
WHR: Waist hip-ratio
