# Supplementary material for: The Norwegian dietary guidelines and colorectal cancer survival (CRC-NORDIET) study: a food-based multicentre randomized controlled trial
Source: BMC Cancer. 2017 Jan 30;17:83. doi: 10.1186/s12885-017-3072-4 (PMC5282711; doi:10.1186/s12885-017-3072-4)
Supplement: Additional file 1: — The CRC-NORDIET intervention program (DOC 42 kb) [file 12885_2017_3072_MOESM1_ESM.doc]

**Additional file 1. The CRC-NORDIET intervention program**

*NFBDG: Norwegian food-based dietary guidelines

|  | **Intervention group** | **Control group** |
| --- | --- | --- |
| Dietary counselling by a registered clinical dietitian | Individual counseling at all visits at the study center.  Telephone dietary counseling between visits during the first year of intervention.  Telephone dietary counseling once a year during the maintenance period of intervention | No dietary intervention, only general dietary advice as part of the standard care |
| Discount-card | A discount card with 25% discount on all fresh vegetables, fruit, berries and fish and several healthy foods available during the first year of intervention |
| Delivery of free foods items | Delivery of free healthy food items at every visit at study center during the first 12 months of intervention.  Delivery of a box with free food items to their homes two times during the first 12 months of intervention |
| Cooking course | A one-day cooking course arranged by registered dietitians following a protocol in accordance with the NFBDG |
| CRC-NORDIET webpage | A log-in restricted dynamic webpage containing extensive information regarding the NFBDG, dietary advice, recipes, week menus and portion sizes.  The webpage is available to the end of study and it is continuously updated with new recipes in accordance to the NFBDG |
| Inspiration days | The first inspiration day will contain:  - Lectures about the NORDIET study  - Diet: practical demonstrations of foods (fruits, vegetables, whole-grain products) and portion sizes according to the NFBDG performed by registered clinical dietitians  - Physical activity: Lecture and practical demonstration by the physical therapists of home-based exercises to incorporate in daily life  The patients are invited to inspiration group meetings every year after the first year of intervention. The group meetings will particularly focus on foods dampening oxidative stress and inflammation, and to encourage the patients to continue following the NFBDG. The physical activity section will consist of different topics at each meeting. | The first inspiration day will contain:  - Lectures about the NORDIET study  - Physical activity: Lecture and practical demonstration by the physical therapists of home-based exercises to incorporate in daily life |
| Physical exercise | All the participants are offered free access to exercise facilities at “Pusterommet” (http://pusterommene.no/) during the first year of intervention. They also get individual counseling by a physiotherapist | |
