# Supplementary material for: The Norwegian dietary guidelines and colorectal cancer survival (CRC-NORDIET) study: a food-based multicentre randomized controlled trial
Source: BMC Cancer. 2017 Jan 30;17:83. doi: 10.1186/s12885-017-3072-4 (PMC5282711; doi:10.1186/s12885-017-3072-4)
Supplement: Additional file 2: — Summary of the 13 recommendations of the Norwegian food-based dietary guidelines (NFBDG) (directly translated from the NFBDG) (DOCX 23 kb) [file 12885_2017_3072_MOESM2_ESM.docx]

**Additional file 2. Summary of the 13 recommendations of the Norwegian food-based dietary guidelines (NFBDG) (translated from the NFBDG)**

|  | **Recommendation** | **Explanations to the recommendations** |
| --- | --- | --- |
| 1 | A primarily plant-based diet is recommended, including plenty of vegetables, fruit, berries, wholegrain and fish, and limited quantities of red and processed meat, salt, added sugar and energy-rich foods | - A varied diet is the best way to achieve favourable health effects and an optimum intake of nutrients. - Choose mainly foods that contain limited quantities of fat, sugar and salt. - Choose foods that help to ensure an adequate intake of nutrients. |
| 2 | It is recommended to maintain a balance between energy intake and energy expenditure | - Energy intake from foods and drinks and energy consumption through physical activity, should be balanced, so that weight is maintained within the normal range. - Regular physical activity helps to maintain the energy balance. A large proportion of the population is overweight. For the overweight, weight loss should combine more physical activity with an energy-reduced diet. - The consumption of foods with high energy content should be limited. - The consumption of drinks with added sugar, such as carbonated drinks should be limited. - Within each food group, it is recommended to choose products that have the Keyhole label |
| 3 | Eat at least five portions of vegetables, fruit and berries every day | - It is recommended to eat at least five portions, corresponding to at least 500 grams altogether, of vegetables, fruit and berries every day. - About half of this intake should be in the form of vegetables and about half fruit and berries. - A portion corresponds to about 100 grams, for example as mixed salad, carrot, broccoli or cauliflower as an accompaniment to a main meal, a medium sized piece of fruit (apple, pear or orange) or a small bowl of berries. As a maximum, one glass of juice can be included as one portion. - The recommendation is to eat a variety of vegetables, fruit and berries of different colours, and include tomatoes and vegetables in the onion family in the diet. - Fresh, tinned, frozen, raw and cooked vegetables, fruit and berries can all be included. Dried fruit can also be included, but the portion size should be adjusted downward, and products with no added sugar should be chosen. - It is recommended to consume a moderate amount of nuts (about 140 grams per week). The nuts should be unsalted. The nuts are in addition to the recommended five portions of vegetables, fruit and berries. Nuts have high energy content and a high intake can promote weight increase. - Potatoes are not included in the recommended five portions of vegetables, fruit and berries. Potatoes are however an important food in the Norwegian diet and can certainly be included in a varied diet. Potatoes have a higher content of dietary fibre and more vitamins and minerals per energy unit that ordinary rice or pasta. Choose boiled or baked potatoes rather than chips, crisps and other potato products with added fat and sugar. - Pulses, seeds, spices and herbs are not included in the recommended five portions of vegetables, fruit and berries. These foods do however often have a high nutrient content and can certainly be included in a varied diet. |
| 4 | Eat at least four portions of wholegrain products every day | - Four portions of wholegrain products corresponds to about 70-90 grams of wholegrain per day (75 g of wholegrain per 10 MJ (2.400 kcal)). - Three slices of wholemeal bread or a large portion of wholemeal pasta or brown rice all correspond to about 75 grams of wholegrain. Breakfast cereals, porridge and crisp bread made with wholegrain are also good wholegrain sources. - At least half of the total consumption of grain products should be in the form of wholegrain. - Preferably, choose grain products with a high fibre content and low content of sugar, fat and salt, such as Keyhole-labelled products and wholemeal breads - Limit the consumption of grain products with a high content of fat, salt and sugar, such as a number of types of cakes, cereals, pizza and snacks. |
| 5 | Eat the equivalent of two or three portions of fish per week | - Weekly consumption of about 300-450 grams of fish is recommended. This corresponds to two or three main meal portions per week. - Alternatively, fish as a main meal can be replaced with fish as a sandwich topping. Six sandwich topping portions of fish approximately correspond to one main meal portion. - Both fatty and lean fish can be included, but it is recommended that at least 200 grams of the intake should be of fatty fish. - Preferably, choose Keyhole-labelled fish products |
| 6 | Low-fat dairy products should be included in your daily diet | - The daily consumption of low-fat dairy products is important for most people in order to ensure an adequate intake of certain nutrients, including calcium and iodine. Low-fat dairy products should therefore be included in the diet. - The consumption of dairy products that contain high levels of saturated fat and/or a high energy content (i.e. more than 950-1,150 kJ or 225-275 kcal per 100 grams), such as full-cream milk, cream, fatty cheese and butter, should be limited. This advice must be seen in context with the other dietary recommendations, so as to ensure a good fat quality in the complete diet. - Preferably, choose Keyhole-labelled dairy products. |
| 7 | It is recommended to eat lean meat and lean meat products, and limit the intake of red meat and processed meat | - Lean meat products are important for most people in order to ensure an adequate consumption of a number of nutrients. Moderate consumption of lean meat products can therefore be included in the diet. - This advice must be seen in context with the other dietary recommendations, so as to ensure a good fat quality in the complete diet. - Choose meat and meat products with a low fat and salt content. Preference should be given to the consumption of unprocessed meat. - Limit the consumption of red meat (beef, pork, lamb and goat) to 500 grams per week. This corresponds to two main meals with red meat and a limited amount as sandwich topping a week. When reducing the consumption of red meat, preference should be given to cutting the consumption of processed red meat. - Those with a high consumption of red meat could preferably replace some of this with white meat and fish. - Consumption of processed meat products (smoked, salted or preserved with nitrate or nitrite) should be limited. - Choosing Keyhole-labelled meat and meat products is recommended. |
| 8 | It is recommended to use cooking oil, liquid margarine or soft margarine | - Cooking oils and margarine with a low content of saturated fatty acids and a high content of unsaturated fatty acids, such as plant oils (e.g. rapeseed, sunflower, olive and soya oils) and liquid or soft margarine, should be used in preference to similar products with a great proportion of saturated fatty acids (such as those that contain a high percentage of palm oil) and low proportion of unsaturated fatty acid. - Limit the use of butter and butter-margarine blends because these have a high content of saturated fatty acids and a low content of polyunsaturated fatty acids. Butter and animal fats also may contain trans fatty acids and cholesterol. - The consumption of foods with high energy content should be limited. Cooking oils and soft or liquid margarine have high energy content, but also contribute with polyunsaturated fatty acids and fat soluble vitamins, and should therefore be included in the diet. - This advice must be seen in context with the other dietary recommendations, so as to ensure a good fat quality in the complete diet. |
| 9 | Water is recommended as the primary choice of drinks | - It is recommended that water makes up the major part of the fluid requirement. This includes tap water and bottled and mineral water (only normal mineral water not sweetened and carbonated soft drinks). - Tap water and most types of mineral water contain insignificant amounts of sodium (salt), but some types of mineral water can contain significant amounts of sodium (see Recommendation 11). - Skimmed milk and extra low-fat milk can certainly be included as a drink in the diet, so as to ensure an adequate intake of calcium and iodine (see Recommendation 6). - Consumption of alcohol is not recommended. - The consumption of drinks with added sugar, such as carbonated drinks should be limited (see Recommendations 2 and 10). - Fruit juice may be included as part of the recommendation for fruit, berries and vegetables (see Recommendation 3). High consumption of fruit juice should however be avoided. - Consumption of acidic (low pH) drinks, such as carbonated drinks with sugar or artificial sweeteners, and juice should be limited outside from mealtimes. |
| 10 | Limit your intake of added sugar | - It is recommended that the intake of added sugar should be limited to less than 10% of the total energy intake. - It is recommended to reduce the consumption of carbonated drinks, soft drinks, nectar, sweet biscuits, cakes and sweets. - The consumption of drinks with added sugar should be limited (see Recommendations 2 and 9). |
| 11 | Limit your intake of salt | - It is recommended to limit the intake of salt (sodium chloride) to a maximum of 6 grams per day (which corresponds to 2.4 grams of sodium). - Preferably, choose food with a low salt content. If food products state salt content, choose products with a low salt content or those with the Keyhole label. - Limit the consumption of food products with a high salt content. Industrial and processed food products contribute 70-80% of salt consumption for most people. Non-processed food contains far less salt than most processed food products. - Limit the use of table salt and salt in the preparation of food. Use other flavourings such as herbs and salt-free spices instead of salt. - Limit the consumption of mineral water with high levels of sodium or a high salt content. Tap water contains insignificant amounts, while mineral water may contain a considerable amount (1 gram of salt per litre, i.e. 0.4 grams of sodium per litre). |
| 12 | Dietary supplements may be necessary to ensure an adequate intake of nutrients for some groups in the population | - Dietary supplements are unnecessary for most people if they have a varied and healthy diet. - If a deficiency of a nutrient is clinically documented, a dietary supplement may be a good alternative if a corresponding intake from foods is difficult. This applies for example to iron deficiency, which is not uncommon among women. Iron supplements are not recommended as a general preventive measure, only if iron deficiency anaemia or low iron status has been documented. - Persons who do not eat fatty fish or who have an intake lower than the recommended lower limit (i.e. 200 grams per week) should take a daily supplement of cod liver oil or other omega-3 supplements, so as to ensure an adequate intake of long-chain polyunsaturated omega-3 fatty acids (EPA, DHA). The primary advice however is to eat fatty fish (see Recommendation 5). - Persons who do not have a sufficient intake of vitamin D should take cod liver oil or another vitamin D supplement daily during the period of the year with little exposure to the sun. Elderly people who spend little time out in the sunlight should take cod liver oil or another supplement with 10 micrograms of vitamin D per day in addition to regular dietary consumption. This also applies to persons with dark skin and others with too low exposure to sunlight. - Persons with a low energy intake (6.5-8 MJ/day or 1.550-1.900 kcal/d), should consider taking a multivitamin and mineral supplement in addition to their regular diet. - Persons with a very low energy intake (less than 6.5 MJ/d or 1.550 kcal/d) should always take a multivitamin and mineral supplement in addition to the regular diet. This applies especially to elderly people with low dietary intakes. - Women of childbearing age are recommended to take a supplement containing 400 micrograms of folate every day for a month before anticipated conception and for the first two or three months of pregnancy. - Care is advised when taking several supplements that contain the same nutrient, since a high intake could have a damaging effect. |
| 13 | It is recommended that everyone participates in least 30 minutes of physical activity per day | - Spend at least 30 minutes a day in moderate physical activity, corresponding to at least a brisk walk. If your general condition allows, this can be increased to an hour or more every day. Generally speaking, any form of physical exercise is better than none. - The time spent in physical activity can be divided into periods during the course of the day. - Physical activity is favourable for weight reduction and for prevention of weight increase after weight reduction. To maintain a large weight loss, 60 to 90 minutes of moderate physical activity most days a week is recommended. |
