# Supplementary material for: The Norwegian dietary guidelines and colorectal cancer survival (CRC-NORDIET) study: a food-based multicentre randomized controlled trial
Source: BMC Cancer. 2017 Jan 30;17:83. doi: 10.1186/s12885-017-3072-4 (PMC5282711; doi:10.1186/s12885-017-3072-4)
Supplement: Additional file 3: — Detailed list of foods and drinks with high contents of redox-active compounds and/or antioxidative effects (DOCX 22 kb) [file 12885_2017_3072_MOESM3_ESM.docx]

**Additional file 3. Detailed list of foods and drinks with high content of redox-active compounds and/or antioxidative effects**

The following foods and drinks have high content of redox-active compounds and/or may have antioxidative effects individually or in combination in vitro models, animal models, clinical trials and/or epidemiological studies: coffee [1-8], green tea [2-4, 6, 9-11], black tea [2-4, 6], onion [1, 12], broccoli [1, 6, 9-11], tomatoes [6, 9-11], red cabbage [2-4, 9-11], kale [9-11], Brussel sprouts [9-11], artichoke [2-4], curly kale [2-4], peppers/paprika [2-4], chili peppers [2-4], carrots [9-11], pomegranates [2-4, 9-11], garlic [6], kiwifruit [13, 14], apples [6, 9-11], orange [6, 9-11], grapes [2-4, 9-11], plums [2-4], cherries [9-11], walnuts [2-4, 6, 9-11], chestnuts [2-4], peanuts [2-4], hazel nuts [2-4], almonds [2-4], thyme [1-4, 9-11, 15], oregano [1-4, 9-11, 15], lemon balm [15], clove [2-4, 15], allspice [2-4, 15], peppermint [2-4, 15], sage [2-4, 15], turmeric [1], rosemary [1-4, 9-11, 15], saffron [2-4], estragon [2-4], elderberries [16], dog rose [1-4, 9-11], cinnamon [2-4, 6, 15], chokeberries [9-11], blueberries/bilberries [2-4, 6, 9-11, 16, 17], blackberries [2-4, 9-11, 16], cranberries [2-4, 9-11], strawberries [2-4, 9-11], raspberries [2-4, 9-11], crowberries [2-4], black currants [2-4], dark chocolate [1-4, 9-11], pecan nuts [2-4, 9-11], olive [2-4, 9-11] and barley [2-4].

Furthermore, we have also identified that the following foods and drinks may have anti-inflammatory effects individually or in combination in cell cultures, animal models, clinical trials and/or epidemiological studies: coffee [5, 18-22], tomatoes [18, 21], carrots [21], pomegranates [21], walnuts [21, 22], nuts [23], strawberries [21], blueberries/bilberries [24, 25], crowberries [21], blackberries [21], dog rose [14], whole grains [26], thyme [21, 22], oregano [21, 22], turmeric [21], clove [21], allspice [21] and rosemary [21].

**List of references for additional file 3:**

1. Balstad TR, Carlsen H, Myhrstad MC, Kolberg M, Reiersen H, Gilen L, Ebihara K, Paur I, Blomhoff R: Coffee, broccoli and spices are strong inducers of electrophile response element-dependent transcription in vitro and in vivo - studies in electrophile response element transgenic mice. Mol Nutr Food Res 2011;55(2):185-97.

2. Carlsen MH, Halvorsen BL, Holte K, Bohn SK, Dragland S, Sampson L *et al*: The total antioxidant content of more than 3100 foods, beverages, spices, herbs and supplements used worldwide. Nutr J 2010;9:3.

3. Halvorsen BL, Carlsen MH, Phillips KM, Bohn SK, Holte K, Jacobs DR, Jr., Blomhoff R: Content of redox-active compounds (ie, antioxidants) in foods consumed in the United States. Am J Clin Nutr 2006;84(1):95-135.

4. Halvorsen BL, Holte K, Myhrstad MC, Barikmo I, Hvattum E, Remberg SF *et al*: A systematic screening of total antioxidants in dietary plants. J Nutr 2002;132(3):461-71.

5. Paur I, Balstad TR, Blomhoff R: Degree of roasting is the main determinant of the effects of coffee on NF-kappaB and EpRE. Free Radic Biol Med 2010;48(9):1218-27.

6. Qureshi SA, Lund AC, Veierod MB, Carlsen MH, Blomhoff R, Andersen LF, Ursin G: Food items contributing most to variation in antioxidant intake; a cross-sectional study among Norwegian women. BMC Public Health 2014;14:45.

7. Russnes KM, Wilson KM, Epstein MM, Kasperzyk JL, Stampfer MJ, Kenfield SA *et al*: Total antioxidant intake in relation to prostate cancer incidence in the Health Professionals Follow-Up Study. Int J Cancer 2014;134(5):1156-65.

8. Svilaas A, Sakhi AK, Andersen LF, Svilaas T, Strom EC, Jacobs DR, Jr., Ose L, Blomhoff R: Intakes of antioxidants in coffee, wine, and vegetables are correlated with plasma carotenoids in humans. J Nutr 2004;134(3):562-7.

9. Bohn SK, Myhrstad MC, Thoresen M, Holden M, Karlsen A, Tunheim SH *et al*: Blood cell gene expression associated with cellular stress defense is modulated by antioxidant-rich food in a randomised controlled clinical trial of male smokers. BMC Med 2010;8:54.

10. Brevik A, Karlsen A, Azqueta A, Tirado AE, Blomhoff R, Collins A: Both base excision repair and nucleotide excision repair in humans are influenced by nutritional factors. Cell Biochem Funct 2011;29(1):36-42.

11. Karlsen A, Svendsen M, Seljeflot I, Sommernes MA, Sexton J, Brevik A *et al*: Compliance, tolerability and safety of two antioxidant-rich diets: a randomised controlled trial in male smokers. Br J Nutr 2011;106(4):557-71.

12. Myhrstad MC, Carlsen H, Nordstrom O, Blomhoff R, Moskaug JO: Flavonoids increase the intracellular glutathione level by transactivation of the gamma-glutamylcysteine synthetase catalytical subunit promoter. Free Radic Biol Med 2002;32(5):386-93.

13. Brevik A, Gaivao I, Medin T, Jorgenesen A, Piasek A, Elilasson J *et al*: Supplementation of a western diet with golden kiwifruits (Actinidia chinensis var.'Hort 16A':) effects on biomarkers of oxidation damage and antioxidant protection. Nutr J 2011;10:54.

14. Karlsen A, Svendsen M, Seljeflot I, Laake P, Duttaroy AK, Drevon CA, Arnesen H, Tonstad S, Blomhoff R: Kiwifruit decreases blood pressure and whole-blood platelet aggregation in male smokers. J Hum Hypertens 2013;27(2):126-30.

15. Dragland S, Senoo H, Wake K, Holte K, Blomhoff R: Several culinary and medicinal herbs are important sources of dietary antioxidants. J Nutr 2003;133(5):1286-90.

16. Carlsen H, Myhrstad MC, Thoresen M, Moskaug JO, Blomhoff R: Berry intake increases the activity of the gamma-glutamylcysteine synthetase promoter in transgenic reporter mice. J Nutr 2003;133(7):2137-40.

17. Myhrstad MC, Carlsen H, Dahl LI, Ebihara K, Glemmestad L, Haffner K, Moskaug JO, Blomhoff R: Bilberry extracts induce gene expression through the electrophile response element. Nutr Cancer 2006;54(1):94-101.

18. Andersen LF, Jacobs DR, Jr., Carlsen MH, Blomhoff R: Consumption of coffee is associated with reduced risk of death attributed to inflammatory and cardiovascular diseases in the Iowa Women's Health Study. Am J Clin Nutr 2006;83(5):1039-46.

19. Kolberg M, Paur I, Balstad TR, Pedersen S, Jacobs DR, Jr., Blomhoff R: Plant extracts of spices and coffee synergistically dampen nuclear factor-kappaB in U937 cells. Nutr Res 2013;33(10):817-30.

20. Kolberg M, Pedersen S, Mitake M, Holm KL, Bohn SK, Blomhoff HK, Carlsen H, Blomhoff R, Paur I: Coffee inhibits nuclear factor-kappa B in prostate cancer cells and xenografts. J Nutr Biochem 2015.

21. Paur I, Austenaa LM, Blomhoff R: Extracts of dietary plants are efficient modulators of nuclear factor kappa B. Food Chem Toxicol 2008;46(4):1288-97.

22. Paur I, Balstad TR, Kolberg M, Pedersen MK, Austenaa LM, Jacobs DR, Jr., Blomhoff R: Extract of oregano, coffee, thyme, clove, and walnuts inhibits NF-kappaB in monocytes and in transgenic reporter mice. Cancer Prev Res (Phila) 2010;3(5):653-63.

23. Blomhoff R, Carlsen MH, Andersen LF, Jacobs DR, Jr.: Health benefits of nuts: potential role of antioxidants. Br J Nutr 2006;96 Suppl 2:S52-60.

24. Karlsen A, Paur I, Bohn SK, Sakhi AK, Borge GI, Serafini M *et al*: Bilberry juice modulates plasma concentration of NF-kappaB related inflammatory markers in subjects at increased risk of CVD. Eur J Nutr 2010;49(6):345-55.

25. Karlsen A, Retterstol L, Laake P, Paur I, Bohn SK, Sandvik L, Blomhoff R: Anthocyanins inhibit nuclear factor-kappaB activation in monocytes and reduce plasma concentrations of pro-inflammatory mediators in healthy adults. J Nutr 2007;137(8):1951-4.

26. Jacobs DR, Jr., Andersen LF, Blomhoff R: Whole-grain consumption is associated with a reduced risk of noncardiovascular, noncancer death attributed to inflammatory diseases in the Iowa Women's Health Study. Am J Clin Nutr 2007;85(6):1606-14.
