# Supplementary material for: The Norwegian dietary guidelines and colorectal cancer survival (CRC-NORDIET) study: a food-based multicentre randomized controlled trial
Source: BMC Cancer. 2017 Jan 30;17:83. doi: 10.1186/s12885-017-3072-4 (PMC5282711; doi:10.1186/s12885-017-3072-4)
Supplement: Additional file 4: — Questionnaires, biological samplings and measurements (DOC 43 kb) [file 12885_2017_3072_MOESM4_ESM.doc]

**Additional file 4. Questionnaires, biological samplings and measurements**

|  | **Baseline** | **6 mths** | **12 mths** | **3 yrs** | **5 yrs** | **7 yrs** | **10 yrs** | **15 yrs** |
| --- | --- | --- | --- | --- | --- | --- | --- | --- |
| *Demographic information* | X | X | X | X | X | X | X | X |
| *Assessment of dietary intake*  FFQ  Compliance questionnaire  Food records  24-hr recall | X  X  X  X | X  X | X  X | X  X | X  X | X  X | X  X | X  X |
| *Assessment of physical activity and function*  PA monitor  Self-reported PA  6MWT  Sit-to-stand test  Hand grip strength | X  X  X  X  X | X  X  X  X  X | X  X  X  X  X | X  X  X  X  X | X  X  X  X  X | X  X  X  X  X | X  X  X  X  X | X  X  X  X  X |
| *Assessment of nutritional status*  PG-SGA | X | X | X | X | X | X | X | X |
| *Anthropometric measurements*  Body weight  Height  Waist and hip circumference | X  X  X | X  X  X | X  X  X | X  X  X | X  X  X | X  X  X | X  X  X | X  X  X |
| *Body composition analysis*  BIA  DXA  CT* | X  X | X  X | X  X | X  X | X  X | X  X | X  X | X  X |
| *Blood pressure* | X | X | X | X | X | X | X | X |
| *Biological samples*  Venous blood samples  Buffy coats from EDTA  Dried blood spot samples  Urine samples  Feces samples  Tumor tissue** | X  X  X  X | X  X  X | X  X  X | X  X  X  X  X | X  X  X | X  X  X  X  X | X  X  X | X  X  X  X  X |
| *Oral glucose tolerance test* | X | X | X | X | X | X | X | X |
| *Health status*  Quality of life  Fatigue | X  X | X  X | X  X | X  X | X  X | X  X | X  X | X  X |
| *CT images are routinely taken for clinical purposes pre-surgery and 5 and 10 years after surgery.  **Tumor tissue will be collected at surgery  FFQ: Food frequency questionnaire; 6MWT: 6 minutes walking test; BIA: bioelectrical impedance analysis; CT: computerized tomography; DXA: dual-energy x-ray absorptiometry; EDTA: ethylenediaminetetraacetic acid; PG-SGA: Patient-Generated Subjective Global Assessment; PA: physical activity | | | | | | | | |
